# Probiotics and eating disorders: a systematic review of humans and animal model studies

**DOI:** 10.1186/s40337-024-01143-4

**Published:** 2024-11-25

**Authors:** Hossein Bahari, Camellia Akhgarjand, Seyedeh Nooshan Mirmohammadali, Mahsa Malekahmadi

**Affiliations:** 1 Transplant Research Center, Clinical Research Institute, Mashhad University of Medical Sciences, Mashhad, Iran; 2 Department of Clinical Nutrition, School of Nutritional Sciences and Dietetics, Tehran University of Medical Sciences, Tehran, Iran; 3 Department of Food, Nutrition, Dietetics and Health, Kansas State University, Manhattan, KS United States; 4 Imam Khomeini Hospital Complex, Tehran University of Medical Sciences, Tehran, Iran

**Keywords:** Probiotics, Prebiotics, Gut microbiota, Eating disorders, Mental health, Systematic review

## Abstract

**Background:**

Eating disorders are complex mental health conditions that significantly impact physical and mental well-being. Current research suggests a potential link between eating disorders and the gut microbiota, highlighting the role of gut-brain communication and its influence on nutrient absorption. Probiotics, which are beneficial bacteria, have shown promise in modulating the gut microbiota and may offer complementary interventions in the treatment of eating disorders.

**Methods:**

A comprehensive search was conducted in electronic databases, including PubMed/Medline, Scopus, and Web of Science, from inception to January 2024 to analyze the existing literature on the effects of probiotic supplementation in eating disorders. The search strategy included terms related to probiotics, prebiotics, eating disorders, and food addiction. The human studies were assessed for risk of bias using the Cochrane tool. The quality of animal studies was evaluated using the risk of bias (RoB) tool from the Systematic Review Centre for Laboratory Animal Experimentation.

**Results:**

Of the 417 papers, 12 eligible studies were included comprising five animal and seven clinical studies. Clinical trials ranged from 10 to 20 weeks and were randomized and parallel-arm design. The included studies varied in terms of sample characteristics, intervention types, and outcome measures. Preliminary findings suggest that probiotics may influence gut microbiota composition and may offer support in the treatment of eating disorders.

**Conclusions:**

The reviewed studies showed that probiotic supplementation may have a role in reducing food addiction and binge eating, and enhancing satiety, regulating food intake as well as positively affecting mood. However, further studies with better quality and larger sample size are needed to further validate these findings.

**Supplementary Information:**

The online version contains supplementary material available at 10.1186/s40337-024-01143-4.

## Abstract (plain language summary)

Eating disorders are serious mental health conditions that impact both physical and mental well-being. Recent studies have explored a potential link between eating disorders and the gut microbiota, which is involved in the communication between the brain and gut, as well as nutrient absorption. Probiotics, which are beneficial bacteria, have shown promise in helping to balance the gut microbiota and may offer support in the treatment of eating disorders. Research has examined the effects of probiotics on eating disorders, and while the studies vary in design and outcomes, they suggest that there may be an effect of probiotics on gut microbiota and may have some benefits in this context. However, the current paper has identified a small number of animal and human studies, so further studies are required to validate these findings.

**Supplementary Information:**

The online version contains supplementary material available at 10.1186/s40337-024-01143-4.

## Introduction

An eating disorder is a mental health condition marked by abnormal eating habits that have profound effects on both physical and mental well-being. These disorders encompass intense emotions, attitudes, and behaviors related to food and body weight, with serious consequences such as food addiction, nutritional deficiencies, electrolyte imbalances, elevated risk of suicide attempts, mortality, and reduced quality of life compared to the general population and other psychiatric conditions [1, 2]. Specific types include Anorexia Nervosa, characterized by a fear of weight gain and restrictive eating; Bulimia Nervosa, involving binge eating followed by compensatory behaviors; Binge Eating Disorder (BED), featuring episodes of consuming large amounts of food without compensatory actions; and Avoidant/Restrictive Food Intake Disorder (ARFID), marked by limited food preferences leading to inadequate nutrition and food addiction, a behavioral disorder characterized by an uncontrollable urge to consume certain foods, often high in sugar, fat, or salt, despite negative consequences to one's health or well-being. Each type poses unique challenges, emphasizing the importance of comprehensive treatment approaches [3, 4]. Over the last two decades, there has been a substantial enhancement in our comprehension of the prevalence and consequences of eating disorders. In Western settings, a notable proportion of young individuals have reported the presence of an eating disorder. Specifically, early adulthood sees a prevalence ranging from 5.5% to 17.9% among young women and 0.6% to 2.4% among young men according to the DSM-5 criteria [5]. Within the spectrum of highly consequential psychiatric disorders, eating disorders (EDs) contribute to an annual mortality toll of 10,200 individuals in the United States (U.S.) exclusively. Moreover, these disorders exhibit an astonishing lifetime prevalence, impacting a substantial 28.8 million Americans [6]. Notably, individuals identifying as gender and sexual minorities were found to be at a particularly elevated risk [7]. The economic burden attributed to eating disorders was projected to amount to $64.7 billion during the fiscal year 2018-2019, with an accompanying per-person cost estimated at $11,808. This underscores the considerable financial impact of eating disorders and emphasizes the need for comprehensive strategies to mitigate their economic repercussions [8].

Eating disorder treatments include psychotherapy, nutritional counseling, and medical monitoring. Medications, support groups, and, in severe cases, inpatient or residential treatment may also be utilized. A personalized and multidisciplinary approach is crucial for effective treatment, emphasizing early intervention for better outcomes [9]. Research indicates a link between eating disorders and the gut microbiota, the microorganisms in the digestive tract. Changes in gut bacteria, influenced by factors like diet and stress, may affect mental health through the gut-brain axis [10]. The gut-brain axis is a bidirectional communication system between the gut and the brain, involving neural, hormonal, and immune pathways. It influences various physiological processes, including digestion, mood regulation, and immune responses, with disruptions implicated in conditions like IBS and psychiatric disorders [11]. While recognized, further research is needed to understand and potentially leverage this connection for treatment [12].

Gut microbiota refers to the diverse community of microorganisms, including bacteria, viruses, fungi, and other microbes, residing in the gastrointestinal tract, particularly the stomach and intestines which are characterized based on alpha and beta diversity. Alpha diversity assesses diversity within a single community, while beta diversity evaluates diversity between different communities. Both measures are essential in understanding the complexity and variability of ecosystems [13]. The composition and balance of the gut microbiota are influenced by factors like diet, medications, and environmental exposures [14]. Research suggests that the gut microbiota also has implications for overall health, including its potential influence on mental health including eating disorders and conditions such as obesity and inflammatory diseases [15]. Prebiotics are non-digestible compounds in certain foods that nourish beneficial gut bacteria, promoting their growth. Probiotics, found in fermented foods and supplements, are live microorganisms that contribute to a balanced gut microbiota, conferring health benefits. Synbiotics combine prebiotics and probiotics to create a synergistic effect, enhancing the survival and effectiveness of beneficial microorganisms in the gut. This combination supports a healthy gut environment and overall digestive health [16].

Scientific evidence underscores the role of dysbiosis, an alteration in normal microbial composition, in the development of eating disorders, particularly when coupled with specific genetic susceptibilities [17]. An imbalanced gut microbiota induces changes in gut permeability, leading to an increased translocation of microbial metabolites, known as endotoxemia, from the lumen of the gastrointestinal tract to adjacent tissues and eventually to the systemic circulation. These metabolites can signal the brain to modulate host behavior, which may explain the link between many central nervous system disorders and a compromised gut barrier [18]. Currently, therapeutic approaches targeting microbiota correction include fecal microbiota transplantation (FMT) [10], and there is also consideration for the use of prebiotics and probiotics to address microbiota alterations [19]. Recent research and systematic reviews have highlighted gut dysbiosis as a potential hallmark in anorexia nervosa, suggesting promising therapeutic targets [20]. Previous systematic reviews concluded that maintaining microbiota homeostasis is crucial for a healthy bidirectional communication network between the gut and the brain. Dysbiosis may induce intestinal inflammation, disrupt gut permeability, and activate immune responses in the hypothalamic centers regulating hunger and satiety, thereby contributing to the pathophysiological development of eating disorders. Restoring microbial balance represents a potential therapeutic target for the treatment of eating disorders [12, 21]. Previous systematic reviews were mainly focused on one eating disorder, particularly AN [22, 23]. However, this systematic review evaluates the impact of probiotics on all types of eating disorders.

Given the gap in the current literature, this review is aimed at conducting an updated and thorough analysis of the existing literature to elucidate the potential implications of microbiota alterations in etiopathogenesis and therapeutic strategies through probiotic supplementation for food addiction and eating disorders.

## Materials and methods

The recent Preferred Reporting Items for Systematic Reviews & Meta-Analyses (PRISMA) statement was applied as a framework for reporting this systematic review [24]. A narrative synthesis of the data was conducted without meta-analysis because of the heterogeneity in outcomes and measures, in accordance with the Synthesis Without Meta-analysis guideline [25].

### Search strategy

We chose the search terms based on Medical Subject Headings (MeSH) categories from the National Institutes of Health (NIH), all of which fall under the eating disorders category. The following electronic databases were searched from inception to January 2024 to identify related papers to be entered in this review: PubMed/ Medline, Scopus, and Web of Science (ISI). No date restrictions were imposed. The combination of MESH and non-MESH terms were used for the search, as follows: ("probiotic" OR "probiotics" OR "prebiotic” OR “prebiotics” OR “synbiotic” OR “synbiotics” OR “lactobacillus” OR “bifidobacterium” OR “fermented foods”) AND (“orthorexia nervosa” OR “night eating syndrome” OR “bulimia nervosa” OR “anorexia nervosa” OR “appetite disorder” OR “appetite disorders” OR “eating disorders” OR “eating disorder” OR “feeding disorder” OR “feeding disorders” OR “diabulimia” OR “food addiction” OR “binge eating” OR “pica” OR “Rumination disorder” OR “avoidant-restrictive food intake disorder” OR “arfid” OR “Purging disorder”). A hand search through the reference list of all included trials was also performed to prevent any miss.

### Study selection and eligibility criteria

Two reviewers (H.B and C.A) separately and in duplicate screened the titles/abstracts of all pertinent papers following pre-specified eligibility criteria. Furthermore, full texts were also examined to discover potentially relevant studies. Disagreements were resolved through consensus with the assistance of a third reviewer (M.M). Inclusion criteria for both animal and clinical studies included subjects or animals with eating disorders and intervention with probiotics or fermented foods that naturally contain probiotics or have added probiotics. Also, studies that examined the effects of probiotics on disordered eating behaviors, including binge eating and food addiction, were included. The exclusion criteria in the current systematic review were a) in-vitro studies and b) those with a cross-sectional, cohort, and case-control design, ecological studies, and review articles. This systematic review also excluded studies that had no available full text (for instance, abstract conference papers). Only research articles that were published in the English language were included in this review.

### Data extraction and quality assessment

Data extracted from the eligible studies included first author’s name, publication date, country, study design, sample characteristics (gender, age, BMI, and health status), type and dose of intervention, duration of intervention, sample size, and main outcome measures. Data were independently extracted by two researchers (H.B and C.A) based on the inclusion and exclusion criteria. Disagreements were resolved by discussion between the reviewers and/or by consulting with the third scholar (M.M) when necessary.

The quality of all included studies was independently evaluated by two researchers (C.A and M.M). The quality of all animal interventions was evaluated using the Risk of Bias (RoB) tool from the Systematic Review Centre for Laboratory Animal Experimentation, which is derived from the Cochrane RoB tool and specifically tailored for assessing animal intervention studies [26]. This tool assesses quality based on 10 questions related to six types of bias: selection bias, performance bias, detection bias, attrition bias, reporting bias, and other biases. For all clinical interventions, quality was assessed using the Cochrane Collaboration Risk of Bias Tool [27]. This risk of bias tool covers six domains of bias: selection, performance, detection, attrition, reporting, and other biases. Within each domain, assessments were made based on different items covering different aspects of the domain. We ranked the risk of bias using the proportion of information provided by the eligible studies at low, unclear, or high risk of bias for each domain of the tool. The general risk of bias was considered as high if there were high risk of bias in ≥2 items or unclear risk of bias in ≥3 criteria. Due to the heterogeneity of the outcomes examined in the studies, quantitative analysis was not possible.

## Results

**Fig. 1:**
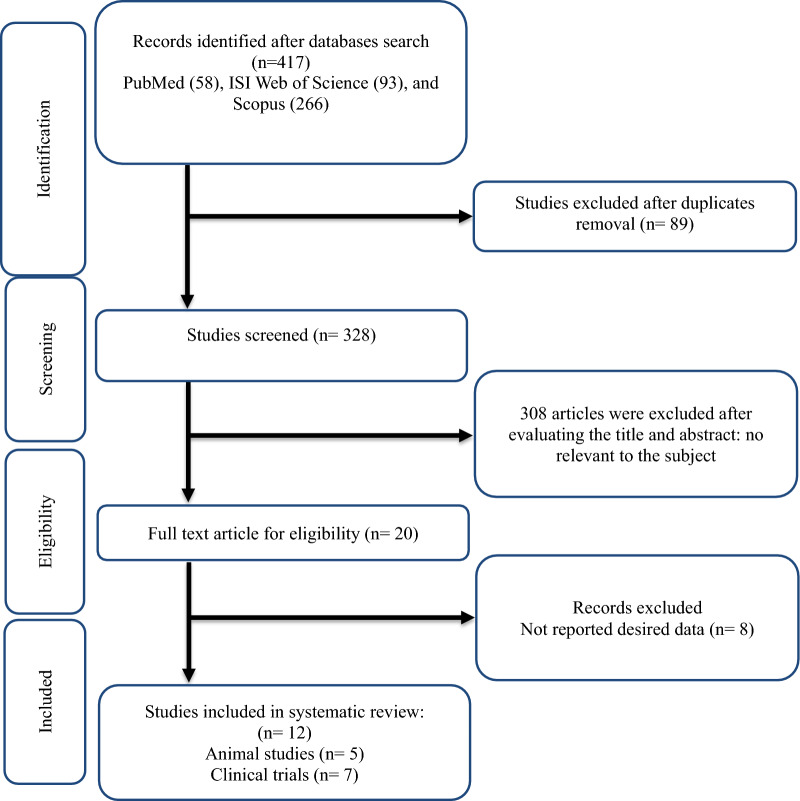
Flow chart of study selection for inclusion trials in the systematic review

As shown in Figure 1, After 417 studies were obtained by initial search, 89 duplicate papers were removed. Among the remaining 328 studies that were screened based on their titles and abstracts, 308 did not meet the inclusion criteria for this review. The full text of 20 studies was evaluated, of which eight papers were excluded due to not reporting the desired data. Finally, 12 studies were included in this systematic review. The studies included in this systematic review were divided into two groups: human studies (n=7) and animal studies (n=5). Human studies included three studies on people with anorexia nervosa [28–30], two studies on people living with obesity [31, 32], one on people with overweight [33], and one on subjects after Roux-en-Y gastric bypass surgery [34]. Three animal studies were conducted on rats [35–37], one on mice [38], and one on fish [39].

### Human studies

The systematic review examined seven human studies investigating the effects of probiotics on eating disorders, including anorexia nervosa, binge-eating disorder, and food addiction. Studies were conducted between 2002 and 2023, and intervention durations ranged from 10 to 20 weeks. Five studies were parallel, randomized, placebo-controlled, and double-blind [30–34], and the others were parallel and randomized [28, 29]. Four studies were conducted in women [28–31], and three studies examined both genders [32–34]. The characteristics of the studies are shown in Table 1. Among the included studies, two had a high general risk of bias [28, 29], while the rest had a low general risk of bias. The details of the risk of bias assessment in each subclass are shown in Supplementary Table 1. The Yale Food Addiction Scale (YFAS) [34], Binge Eating Scale (BES) [32, 34], and The Three Factor Eating Questionnaire (TFEQ) [31, 33] were used to evaluate eating disorders.

**Table 1:** Characteristics of the included human studies

Studies	Country	Study design	Participants	Sex	Age (years)	Sample size	Trial duration (week)	Means BMI (kg/m^2^)	Intervention	Outcomes
									Strain (concentration)	
Solis et al. 2002 [29]	Spain	Parallel, R	Adolescent female with anorexia nervosa (AN)	F	12–18	31 Group 1: 16 Group 2: 11	20	Group1: 15 Group 2: 16	Group 1: 375 g yoghurt for 10 weeks, the following 10 weeks 450 g fermented milk Group2: 450 g fermented milk for 10 weeks, the following 10 weeks 375 g yoghurt	IFN-γ, BMI
Nova et al. 2006 [28]	Spain	Parallel, R, C	Patients with AN	F	13–19	16	10	14.94	375 yoghurt containing *L. bulgaricus* and *S.* *thermophilus* (10^7^-10^8^ CFU/ml)	IFN-γ, IL-2, IL-1, IL-6, TNF-α, and blood lymphocyte subsets
Sanchez et al. 2017 [32]	Canada	Parallel, R, PC, DB	Patients with obesity	M/F	18–55	105 M: 45 F: 60	12-week weight-loss period (Phase 1) 12 weeks of weight maintenance (Phase 2)	33.8	Probiotic supplement (Lactobacillus rhamnosus CGMCC1.3724 (LPR)) 1.6× 10^8^ CFU/capsule + 210 mg of oligofructose + 90 mg of inulin, twice daily	Appetite sensations, eating behaviors (food cravings and binge eating), and depression
Zaja et al. 2021 [30]	Croatia	Parallel, R, PC, DB	Patients with AN and constipation	F	10–18	31 Intervention: 15 Placebo: 16	12	14.59	*Lactobacillus reuteri* DSM 17938 (10^8^ CFU/table), twice daily	Constipation, body weight, stool frequency and consistency, relief of dyspepsia, weight gain and recovery of malnutrition and Vitamin D3 levels
Narmaki et al. 2022 [31]	Iran	Parallel, R, PC, DB	Women with obesity and food addiction	F	20–50	52 Intervention: 26 Placebo: 26	12	34.5	Probiotic supplement Lactobacillus acidophilus (1.8 × 109 CFU/capsule), Bifidobacterium bifidum (1.8 × 109 CFU/capsule), Bifidobacterium lactis (1.8 × 109 CFU/capsule), Bifidobacterium longum (1.8 × 10^9^ CFU/capsule), Lactobacillus rhamnosus (1 × 10^9^ CFU/ capsule), Lactobacillus reuteri (1 × 10^9^ CFU/capsule)	Anthropometric measurements, eating behavior and appetite, oxytocin, NPY, Leptin
Carlos et al. 2022 [34]	Brazil	Parallel, R, PC, DB	Subjects after Roux-en-Y gastric bypass surgery	M/F	18–59	69 Intervention: 37 Placebo: 32	12	42.84	*Lactobacillus* *acidophilus* NCFM and *Bifidobacterium lactis* Bi-07(5 × 10^9^ CFU/capsule)	Food addiction and binge eating
Choi et al. 2023 [33]	Canada	Parallel, R, PC, DB	Adults with overweight during weight loss	M/F	18–55	67 Intervention: 37 Placebo: 30	12	32.3	Lacticaseibacillus rhamnosus HA-114 (10 × 10^9^ CFU/capsule)	Appetite sensations, eating behaviors (food cravings and binge eating), and mood-related factors

*DB* double-blinded, *C* controlled, *PC* placebo-controlled, *R* randomized, *F* female, *M* male, *BMI* body mass index, *IFN* interferon, *IL* interleukin, *TNF*, tumor necrosis factor, *NPY* Neuropeptide Y

One study found that probiotic supplementation in bariatric surgery patients showed a significant reduction in food addiction and binge eating behaviors one-year post-surgery, suggesting a beneficial role in managing body weight and related anthropometric measures [34], in addition, a diet-based weight-reducing program with probiotic supplementation improved satiety efficiency and eating behavior traits, contributing to improved disordered eating behaviors during a diet-based weight-loss program [32]. While hormone levels related to appetite and metabolism were not the primary focus of the included studies, the interaction between probiotics and the gut-brain axis was explored. Carlos et al. highlighted the potential of probiotics to modulate the microbiota-gut-brain axis, which could indirectly influence hormone levels associated with eating behaviors [34]. Further research is needed to quantify these effects directly. Probiotic supplementation was consistently associated with improvements in eating behaviors across the reviewed studies. It was reported a significant decrease in the number of symptoms of food addiction and binge eating behaviors in the probiotic group compared to the placebo group [31]. This effect on disordered eating behaviors was observed up to one-year post-surgery [34]. Furthermore, it was noted positive changes in eating behavior traits suggest that probiotics could help regulate food intake and reduce unhealthy eating patterns [28]. The psychosocial impacts of probiotics on individuals with eating disorders were examined in studies. The findings indicated that probiotic supplementation, in conjunction with a structured diet program, could positively influence psychosocial behaviors related to eating, such as reduced anxiety and improved mood [33]. These changes are crucial for the long-term management of eating disorders.

### Animal studies

In this section, five articles were included; the characteristics of these articles are shown in Table 2. The overall bias quality scores were provided in Supplementary Table 2, where the majority of studies received a score of ≥7 out of 10 [35, 37–39], and only one study obtained a score of 5 [36]. The following eating disorders were investigated in animal studies: anorexia nervosa and bulimia [36], anorexia nervosa [37], sugar craving [38], binge eating and anxiety-like behavior [35], and one study examined a type of eating disorder in fish characterized by food aversion and reduced feed intake when switched from their preferred live prey diet to an artificial pellet diet [39].

**Table 2:** Characteristics of the included animal studies

Studies	Country	Animal model	Sample size		Dose	Duration (Days)	Intervention	Outcomes
			IG	CG				
Tennoune et al. 2015 [36]	France	Adult Male and female Wistar rats	12	12	10^8^ cells/mL Luria broth (LB) liquid medium	21	Escherichia coli K12	Body weight, food intake, anti- α-MSH and ACTH immunoglobulin (Ig)G, fecal
Trinh et al. 2023 [37]	Germany	Translational activity-based anorexia (ABA) female Wistar rats	12	12	1 × 10^9^ CFU/ml	48	VSL#3®: *Bifidobacterium breve, Bifidobacterium longum, Bifidobacterium infantis, Streptococcus* *thermophilus, Lactobacillus acidophilus, Lactobacillus plantarum, Lactobacillus paracasei, and Lactobacillus delbrueckii subsp. bulgaricus*	Gut-Associated Lymphatic Tissue
Nicol et al. 2023 [38]	France	C57Bl6 mice	8	8	1 × 10^10^ CFU	27	mix of *L. salivarius (LS)* LS7892 and *L. gasseri (LG)* LG6410	Sugar craving
Chen et al. 2021 [39]	China	Mandarin fish (*Siniperca chuatsi*)	*L. plantarum =270* *L. rhamnosus=270* *C. butyricum= 270*	270	10^8^ colony forming unit/mL to the culture water	28	*Lactobacillus plantarum*, *Lactobacillus rhamnosus* and *Clostridium butyricum*	Eating disorder characterized by food aversion and reduced feed intake when switched from their preferred live bait diet to an artificial pellet diet.
Agusti et al. 2021 [35]	Spain	Wistar rats with food addiction	NR	NR	1 ×10^8^ colony-forming units of *B. uniformis*	18	*Bacteroides uniformis* CECT 7771 (*B. uniformis*)	Binge eating and anxiety-like behavior

*IG* intervention group *CG* control group, *α-MSH* α-melanocyte-stimulating hormone, *ACTH* adrenocorticotropic hormone, *NR* not reported

One study highlights the impact of Bacteroides uniformis CECT 7771 in a rat model of food addiction, showing that probiotic supplementation reduced body weight gain and influenced hormone levels related to appetite and stress [35]. Probiotics showed an effect on key neurotransmitters like dopamine, serotonin, and noradrenaline, which are critical in the regulation of reward and appetite [35]. Additionally, probiotic supplementation helped preserve gut health after chronic starvation, improving gut structure, reducing gut-associated lymphoid tissue, enhancing intestinal permeability, and lowering inflammation—factors relevant to conditions like anorexia nervosa [37]. Probiotics also showed influence on the gut-brain axis, affecting eating patterns, reducing stress-induced sugar cravings, increasing water intake, and showing mild anti-depressive effects, with some strains having sex-specific impacts on feeding behavior [36, 38]. Furthermore, probiotics demonstrated immunomodulatory effects, which may contribute to their positive influence on eating disorders, as evidenced by studies in Mandarin fish where stimulated immune responses correlated with improved eating behaviors [39].

## Discussion

There was a limited number of human and animal studies examining the link between probiotics and disordered eating. Based on the limited evidence, there may be several mechanisms that may explain how probiotics may influence the physiological aspects of eating disorders. Probiotics influence both the physiological and psychological aspects of eating disorders through multiple mechanisms, that interact with the gut-brain axis, hormonal regulation, neurotransmitter production, immune function, and mental health. Here is a detailed explanation of these mechanisms:

### Physiological aspects

#### Gut-brain *axis* modulation

The gut-brain axis is a critical pathway in the regulation of eating behaviors. A human study demonstrated that probiotics play a significant role in modulating this connection. Probiotics can stimulate the vagus nerve, which transmits signals between the gut and brain, influencing hunger, satiety, and stress responses [31]. Particularly for those undergoing bariatric surgery, probiotic supplementation has shown promising results in reducing food addiction and binge eating scores [34]. This suggests that probiotics can be beneficial for improving disordered eating behaviors post-surgery. Also, probiotics produce metabolites such as short-chain fatty acids (SCFAs) that can cross the blood-brain barrier and affect brain function, including appetite regulation and mood [40]. A systematic review study conducted in 2020 on 9 studies that examined the population of microbiota in patients with AN, showed that in these patients, the population of microbial species that produce butyrate decreased, and mucin-degrading species increased [20].

#### Neurotransmitter regulation

Animal studies reinforce these findings by demonstrating that probiotics, like Bacteroides uniformis CECT 7771, can significantly reduce body weight gain and modulate neurotransmitter levels related to reward and appetite, such as dopamine, serotonin, and noradrenaline [35, 37]. These changes suggest that probiotics may influence the brain's reward system and appetite regulation, which are crucial in managing compulsive eating behaviors [35]. Some probiotics can increase levels of gamma-aminobutyric acid (GABA), a neurotransmitter that has calming effects and can reduce stress-related eating [41].

#### Hormonal regulation

A human study showed that probiotics impact hormones that regulate hunger and fullness, influencing eating behaviors. Probiotics can help regulate ghrelin levels, preventing excessive hunger [31]. Also, probiotics can enhance leptin sensitivity, ensuring appropriate responses to satiety signals. Probiotics can increase the production of Peptide YY (PYY) and Glucagon-Like Peptide-1 (GLP-1) and reduce food intake [31]. The inclusion of probiotics in diet-based weight-reducing programs also demonstrated improved satiety and better regulation of food intake, which are critical for maintaining healthy eating patterns [42]. According to the above, the gut microbiome plays a significant role in metabolism and energy balance. Dysbiosis, or an imbalance in the gut microbiota, has been linked to disordered eating behaviors [32].

#### Immune system interaction

The immune system plays a role in signaling the brain about the body's state, including hunger and satiety. Human studies indicated that probiotics reduce systemic inflammation by modulating the immune response. Lower inflammation levels are associated with better mood and reduced risk of depression, which can indirectly affect disordered eating behaviors [32, 33]. Probiotics strengthen the gut barrier, preventing the leakage of inflammatory molecules into the bloodstream, which can disrupt metabolic processes and appetite regulation [28, 29]. The ability of probiotics to reverse the increase in GALT suggests that they can modulate the gut microbiome and reduce inflammation. This indicates that gut microbiota alterations are not only present in AN but are also modifiable [37]. The immunomodulatory effects observed in both rats and Mandarin fish suggest that probiotics can enhance immune responses, which may be linked to their beneficial impact on eating disorders [36, 37, 39].

### Psychological aspects

#### Mood improvement

Mental health significantly influences eating behaviors, animal and human studies showed that probiotics have positive effects on mood. Probiotics can reduce symptoms of anxiety and depression, which are often linked to disordered eating behaviors [32, 33, 35] Improved mental health can lead to better control over eating behaviors. By influencing neurotransmitter production and reducing inflammation, probiotics help lower stress levels, which can reduce stress-related eating and binge episodes [35, 42]. In the 2020 systematic review, 16 studies were reviewed, and the results indicated that alpha diversity and SCFA levels were lower in patients with AN, and emotional symptoms and psychopathology of eating disorders seem to be related to changes in gut microbiota [12].

#### Cognitive function

An animal study showed that probiotics can enhance cognitive function, which may help individuals make better food choices and adhere to healthy eating patterns. Improved executive function helps individuals resist cravings and make healthier food choices [38]. Better cognitive function supports the learning of healthy eating habits and the unlearning of disordered eating behaviors [31, 32]. An umbrella systematic review (2021), which examined three systematic review studies related to AN, revealed that the population of fecal microbiota has an inverse relationship with the severity of the disease [43].

#### Behavioral changes

An animal study showed that probiotics can positively influence behaviors related to eating by improving mood and reducing stress. By modulating dopamine and serotonin pathways, probiotics can reduce the reward-driven compulsion to overeat [35]. Improved mood and reduced anxiety can decrease the tendency to eat in response to emotions rather than hunger [33, 35]. The reduction in compulsive eating and modulation of feeding behavior further support the potential of probiotics in treating eating disorders [34, 39].

#### Strengths and limitations

The current study is a comprehensive systematic review of the effect of probiotic supplements on eating disorders, which has been reviewed in both human and animal studies. However, due to the heterogeneity of the population and outcomes examined in the studies, quantitative analysis was not possible. Most of the included human studies were of low risk of bias, and also most of the included animal studies had high quality scores. Also, due to the small number of studies, it is not possible to draw a definite conclusion.

## Conclusion

Given the small number of studies and heterogeneity, it is difficult to draw major conclusions. A human study showed probiotic supplementation probably, reduced food addiction and binge eating. Also, probiotics may improve satiety, regulate food intake, and positively influence psychosocial behaviors like anxiety and mood. Animal studies showed probiotics like Bacteroides uniformis CECT 7771 may reduce body weight gain, modulate neurotransmitter levels, and influence appetite regulation. Probiotics probably have immunomodulatory effects. More studies are needed to determine the type of microbiota and other possible mechanisms, as well as the effectiveness level, in different populations with different physiological and pathological conditions. Therefore, it is recommended to design both animal and human studies with better quality and larger sample size to draw conclusions.

## Supplementary Information

Additional file 1

## Data Availability

All data generated or analysed during this study are included in this published article and its supplementary information files.
