# Supplementary material for: Probiotics and eating disorders: a systematic review of humans and animal model studies
Source: J Eat Disord. 2024 Nov 25;12:193. doi: 10.1186/s40337-024-01143-4 (PMC11587709; doi:10.1186/s40337-024-01143-4)
Supplement: Supplementary file 1 — Additional file 1 [file 40337_2024_1143_MOESM1_ESM.docx]

**Probiotics and Eating Disorders: A Systematic Review of Humans and Animal Model Studies**

Hossein Bahari ^1^, Camellia Akhgarjand ^2^, Seyedeh Nooshan Mirmohammadali ^3^, Mahsa Malekahmadi ^4^*

1. Transplant Research Center, Clinical Research Institute, Mashhad University of Medical Sciences, Mashhad, Iran. (Email: [baharihossein3@gmail.com](mailto:baharihossein3@gmail.com))
2. Department of Clinical Nutrition, School of Nutritional Sciences and Dietetics, Tehran University of Medical Sciences, Tehran, Iran. (Email: [kameliaakhgarjand@yahoo.com](mailto:kameliaakhgarjand@yahoo.com) )
3. Department of Food, Nutrition, Dietetics and Health, Kansas State University, Manhattan, KS, United States. (Email: [nooshanmir@ksu.edu](mailto:nooshanmir@ksu.edu) )
4. Imam Khomeini Hospital Complex, Tehran University of Medical Sciences, Tehran, Iran.

***Corresponding author:**

**Mahsa Malekahmadi**

Imam Khomeini Hospital Complex, Tehran University of Medical Sciences, Tehran, Iran.

Email: [malekahmadimahsa@gmail.com](mailto:malekahmadimahsa@gmail.com)

**Supplementary Table 1.** Risk of bias assessment for human studies.

| **Study** | **Random sequence generation** | **Allocation concealment** | **Selective reporting** | **Other sources of bias** | **Blinding (participants and personnel)** | **Blinding (outcome assessment)** | **Incomplete outcome data** | **General risk of bias** |
| --- | --- | --- | --- | --- | --- | --- | --- | --- |
| Solis et al. 2002 | L | U | L | L | U | U | L | High |
| Nova et al. 2006 | L | U | L | L | U | U | L | High |
| Sanchez et al. 2017 | L | L | L | L | L | L | L | Low |
| Zaja et al. 2021 | L | L | L | U | L | L | L | Low |
| Narmaki et al. 2022 | L | L | L | L | L | L | L | Low |
| Carlos et al. 2022 | L | L | L | U | L | L | L | Low |
| Choi et al. 2023 | L | L | L | L | L | L | L | Low |

L; low risk of bias; H, high risk of bias; U, unclear risk of bias

General risk of bias is considered as high if there were high risk of bias in ≥2 items or unclear risk of bias in ≥3 criteria.

**Supplementary Table 2.** Risk of bias assessment for animal studies.

| **Study** | **Q1** | **Q2** | **Q3** | **Q4** | **Q5** | **Q6** | **Q7** | **Q8** | **Q9** | **Q10** | **Quality score** |
| --- | --- | --- | --- | --- | --- | --- | --- | --- | --- | --- | --- |
| Tennoune et al. 2015 | NI | PY | NI | PY | NI | NI | NI | Y | Y | Y | 5 |
| Trinh et al. 2023 | Y | Y | PY | Y | NI | Y | NI | Y | Y | Y | 8 |
| Nicol et al. 2023 | Y | Y | PY | PY | NI | NI | NI | Y | Y | Y | 7 |
| Chen et al. 2021 | Y | NI | PY | PY | NI | Y | NI | Y | Y | Y | 7 |
| Agusti et al. 2021 | Y | PY | PY | PY | NI | Y | Y | Y | Y | Y | 9 |

Abbreviations: Y, yes; PY, probably yes; N, no; PN, probably no; NI, no information; NA, not applicable

Quality of included animal studies was assessed using the SYstematic Review Centre for Laboratory animal Experimentation (SYRCLE) RoB tool for animal intervention studies. Q1. Was the allocation sequence adequately generated and applied? Q2. Were the groups similar at baseline or were they adjusted for confounders in the analysis? Q3. Was the allocation adequately concealed? Q4. Were the animals randomly housed during the experiment? Q5. Were the caregivers and/or investigators blinded from knowledge of which intervention each animal received during the experiment? Q6. Were animals selected at random for outcome assessment? Q7. Was the outcome assessor blinded? Q8. Were incomplete outcome data adequately addressed? Q9. Are reports of the study free of selective outcome reporting? Q10. Was the study apparently free of other problems that could result in high risk of bias?
